# Relationship of matrix Gla protein and vitamin K with vascular calcification in hemodialysis patients

**DOI:** 10.1080/0886022X.2019.1650065

**Published:** 2019-09-20

**Authors:** Sonoo Mizuiri, Yoshiko Nishizawa, Kazuomi Yamashita, Kyoka Ono, Takayuki Naito, Chie Tanji, Koji Usui, Shigehiro Doi, Takao Masaki, Kenichiro Shigemoto

**Affiliations:** a Division of Nephrology, Ichiyokai Harada Hospital, Hiroshima, Japan;; b Ichiyokai Yokogawa Clinic, Hiroshima, Japan;; c Ichiyokai Ichiyokai Clinic, Hiroshima, Japan;; d Department of Nephrology, Hiroshima University Hospital, Hiroshima, Japan

**Keywords:** Cardiovascular disease, coronary artery calcium score, hemodialysis, matrix Gla protein, vitamins K

## Abstract

**Objective:** This study evaluated associations of serum matrix Gla protein (MGP), plasma vitamin K1, and plasma vitamin K2 with coronary artery calcium score (CACS) and cardiovascular disease (CVD) in maintenance hemodialysis (MHD) patients.

**Methods:** Subjects comprised 112 MHD patients aged 30–60 years and 40 age-matched healthy subjects. Total MGP, vitamin K1, vitamin K2, and lipid profile were examined in all subjects; other clinical data, medication use, and CACS were assessed only in MHD patients. Determinants of MGP in all subjects were identified by regression analysis. Factors associated with CACS and CVD in MHD patients were identified by regression analysis and logistic analysis, respectively.

**Results:** Lower plasma levels of vitamin K1 corrected for triglycerides [0.39 (0.24–0.70) vs. 0.77 (0.48–1.34) ng/mg, *p* < 0.001], higher frequency of plasma vitamin K2 ≤ 0.05 ng/ml (*p* = 0.23), and higher serum total MGP (288.4 ± 44.2 vs. 159.7 ± 40.6 ng/ml, *p* < 0.0001) were observed in MHD patients than in healthy controls. Total MGP level was significantly associated with levels of vitamin K1 corrected for triglycerides (*p* <0 .001) and vitamin K2 ≤ 0.05 ng/ml (*p* < 0.05) in all subjects. Total MGP level was significantly associated with presence of CVD (*p* <0 .05), but not CACS, in MHD patients.

**Conclusion:** The end-stage renal disease on hemodialysis is a deficiency state of vitamin K. Total MGP was significantly higher in MHD patients compared to healthy subjects and total MGP was associated with the presence of CVD, but not CACS, in MHD patients.

## Introduction

Matrix Gla protein (MGP) is primarily secreted by chondrocytes and smooth vascular muscle cells, and acts as a potent local inhibitor of vascular calcification [1]. However, to be active, MGP must be phosphorylated and carboxylated; such carboxylation is vitamin K-dependent, and phosphorylation is necessary for the secretion of MGP [2]. The vitamin K family includes phylloquinone (vitamin K1) and several menaquinones (vitamin K2) [2–4]. Notably, 72% of patients with chronic kidney disease (CKD) exhibit vitamin K intake lower than recommended levels [5]. Vitamin K status can be quantified by using high-performance liquid chromatography (HPLC) [6,7], a method that requires specific and expensive equipment. It has been suggested that vitamin K-dependent proteins (i.e. plasma abnormal prothrombin, osteocalcin, growth arrest-specific gene-6 protein, and MGP) can be used as indicators of vitamin K status [7]; indeed, previous studies have used these markers to evaluate vitamin K status in hemodialysis (HD) patients [7–10]. A theoretical link exists among MGP, vitamin K, vascular calcification, and cardiovascular disease (CVD); this link is more notable in CKD and HD patients [2,4]. However, atherosclerotic calcification is more prevalent in elderly HD patients; thus, age is a primary risk factor for vascular calcification in such patients [11,12]. Simultaneous assessment of MGP levels, vitamin K levels, and vascular calcification should be performed in age-matched populations. To the best of our knowledge, there are no such studies in the literature. In the present study, we investigated MGP and vitamin K status in age-matched HD patients and healthy controls; in addition, we assessed vascular calcification and CVD in HD patients.

## Materials and methods

### Study population

This cross-sectional study enrolled Japanese 112 maintenance hemodialysis (MHD) patients, 30–60 years of age, who were undergoing regular HD treatment, three sessions per week; concurrently, age-matched Japanese healthy subjects were enrolled. Subjects with a history of neoplastic disease, with active infections, who were receiving anti-vitamin K therapy, or who had undergone an organ transplant were excluded from this study. All procedures performed in studies involving human participants were in accordance with the ethical standards of the institutional and/or national research committee and with the Declaration of Helsinki (as revised in Brazil in 2013). The committee on human research at Ichiyokai Hospital approved the study protocol (authorization no. 201701), and informed consent was obtained from all individual participants included in this study.

### Clinical and biochemical evaluation

Demographic data included age, sex, height, body weight, and body mass index upon study entry in March 2017. For MHD subjects, the following additional data were included: dialysis vintage, original disease, presence of diabetes mellitus, presence of past and present CVD (e.g. coronary artery disease, aortic aneurysms, cerebral infarction, cerebral hemorrhage, and/or peripheral artery disease), presence of hypertension (predialysis blood pressure ≥140/90 mmHg), and medication use. Serum samples from patients were obtained immediately before the first HD session of the week. All serum samples were stored at –80 °C within 30 min of sampling. Serum creatinine, total MGP, total cholesterol, high-density lipoprotein (HDL) cholesterol, low-density lipoprotein (LDL) cholesterol, and triglycerides levels, as well as plasma levels of vitamins K1 and K2, were determined for all subjects. Coronary artery calcium scores (CACS) using the Agatston score [13], based on thoracoabdominal multi-detector computed tomography (MDCT) with an Aquilion 64 TSX-101A (Toshiba Medical Systems, Tokyo, Japan), were solely determined for MHD patients, since the committee on human research at Ichiyokai Hospital did not approve the use of thoracoabdominal MDCT in healthy subjects. Similarly, other laboratory data were solely determined for MHD patients. Both MGP and vitamin K measurements were performed by SRL, Inc. (Tokyo, Japan). Serum total MGP was determined by using enzyme-linked immunosorbent assay (ELISA) kits with the following immunogen: full-length MGP, from Met to Lys103 (SEB477Hu, Cloud-Clone Corp., Houston, TX, USA) [14]. Plasma levels of vitamins K1 and K2 were determined by HPLC with electrochemical detection [15]. A plasma level of vitamin K2 ≤ 0.05 ng/ml cannot be measured. Our hospital laboratory performed all other clinical biochemical analyses.

### Statistical analysis

All statistical analyses were performed with JMP13 (SAS Institute Japan, Tokyo, Japan). The Kolmogorov–Smirnov test was used to determine whether data exhibited a normal distribution. Categorical variables are reported as numbers of patients (percentages); continuous variables are reported as means ± standard deviations (SD) or medians [interquartile ranges (IQRs)], as appropriate. Variables were compared between two groups (healthy controls and MHD patients), or among three groups (MHD patients stratified according to CACS) were compared by the Wilcoxon signed-rank test for continuous variables and Fisher’s exact test for categorical variables. Regression analyses to identify factors associated with serum total MGP levels were performed in all subjects. Regression analyses were performed to identify factors associated with CACS in MHD patients, whereas logistic regression analyses were performed to identify factors associated with the presence of CVD in MHD patients. The distribution of CACS was markedly skewed. Prior to regression analysis and logistic regression analysis, CACS was transformed to Log (CACS + 1) because some study participants exhibited a CACS of 0.

## Results

Demographic laboratory investigation results for the study population are listed in Table 1. There were 152 subjects in all, including 40 healthy controls and 112 MHD patients. The two groups showed no significant differences in age (49 ± 6 years vs. 50 ± 7 years), sex composition, body mass index, or serum triglycerides levels. The serum total cholesterol, HDL cholesterol levels, and LDL cholesterol were significantly lower in MHD patients than in healthy controls (*p* < 0.0001). The median (IQR) serum creatinine levels were 0.73 (0.67–0.81) mg/dl and the mean ± standard deviation eGFR values were 78.2 ± 13.0 ml/min/1.73 m^2^ in healthy controls.

**Table 1.** Demographic data and results of laboratory investigations among study subjects.

Characteristic	All subjects	Healthy subjects	Hemodialysis patients	*p*
	*n* = 152	*n* = 40	*n* = 112	
Age (years)	50 ± 7	49 ± 6	50 ±7	0.10
Male [*n* (%)]	87/152 (57.2)	21/40 (52.5)	66/112 (58.9)	0.57
Body mass index (kg/m^2^)	22.7 (20.2–24.8)	22.8 (20.8–24.7)	22.7 (19.8–25.5)	0.94
Triglycerides (mg/dL)	101 (67–149)	95 (69–161)	102 (64–149)	0.79
Total cholesterol (mg/dL)	170 (143–195)	211 (191–236)	156 (134–176)	<0.0001
HDL cholesterol (mg/dL)	56 (45–71)	69 (56–78)	53 (41–67)	<0.0001
LDL cholesterol (mg/dL)	87 (66–114)	125 (105–143)	80 (62–99)	<0.0001
Serum creatinine (mg/dL)	11.20 (1.25–13.30)	0.73 (0.67–0.81)	12.19 (10.49–13.59)	<0.0001
eGFR (mL/min/1.73 m^2^)		78.2 ± 13.0		

Values are expressed as means ± standard deviations or medians (interquartile ranges), as appropriate.

LDL: low-density lipoprotein, HDL: high-density lipoprotein, eGFR: estimated glomerular filtration rate.

**Figure 1.**
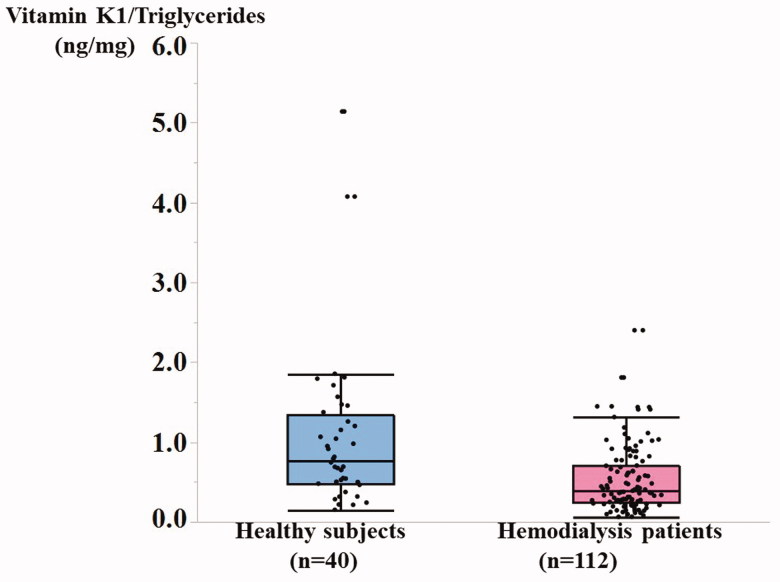
Plasma vitamin K1 levels corrected for triglycerides in hemodialysis patients and healthy controls.

**Figure 2.**
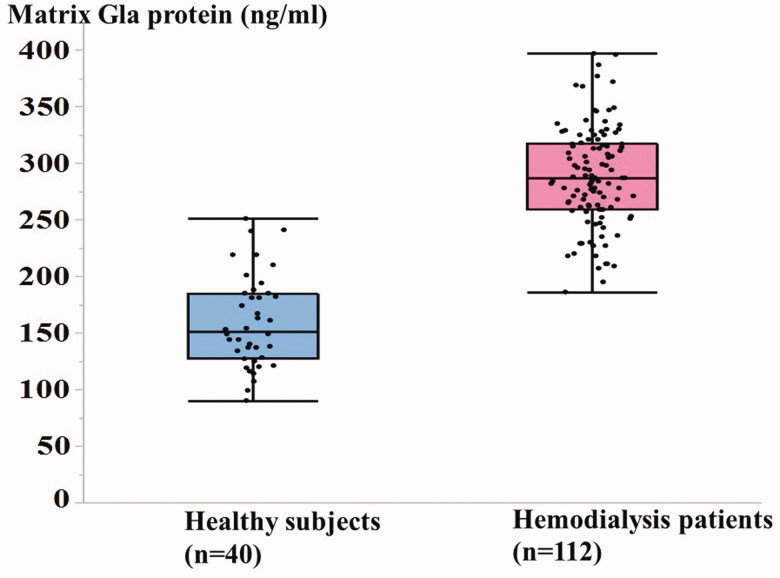
Serum matrix Gla protein levels in hemodialysis patients and healthy controls.

Plasma levels of vitamin K1 alone [0.45 (0.31–0.70) ng/ml vs. 0.87 (0.51–1.31) ng/ml, *p* <0 .0001], and levels of vitamin K1 corrected for triglycerides (vitamin K1/Triglycerides) [0.39 (0.24–0.70) vs. 0.77 (0.48–1.34) ng/mg, *p* < 0.001] were significantly lower in MHD patients (*n* = 112) than in healthy controls (*n* = 40) (Figure 1). The frequency of plasma levels of vitamin K2 ≤ 0.05 ng/ml was higher in MHD patients than in healthy controls, but the difference between the two groups was not significant [88/112 (78.6%) vs. 26/40 (65.0%), *p* = 0 .23]. Among the subjects with measurable plasma levels of vitamin K2, vitamin K2 levels alone [0.09 (0.07–0.14) vs. 0.12 (0.07–0.1) ng/ml, *p* = 0.71] and vitamin K2 levels corrected for triglycerides (vitamin K2/Triglycerides) [0.06 (0.02–0.08) vs. 0.08 (0.04–0.12) ng/mg, *p* = 0.86] were both slightly lower in MHD patients (*n* = 24) than in healthy controls (*n* = 14), but the differences between the groups were not statistically significant. As shown in Figure 2, serum total MGP levels were significantly higher in MHD patients (*n* = 112) than in healthy controls (*n* = 40) (288.4 ± 44.2 vs. 159.7 ± 40.6 ng/ml, *p* <0 .0001).

Regression analyses in all subjects (*n* = 152) are shown in Table 2. Model 1 included age, sex (male), and vitamin K1/Triglycerides, which exhibited significance in univariate analyses. Model 2 was nearly identical to Model 1, but included vitamin K2 ≤ 0.05 ng/ml (unmeasurable low vitamin K2 value) and excluded vitamin K1/Triglycerides. Multivariate analysis showed that the serum total MGP level was significantly associated with age [standardized partial regression coefficient (*β*) 95% confidence interval (CI): 0.31 (1.76–4.79), *p* <0 .0001], sex (male) [*β* (95% CI): 0.27 (10.41–34.06), *p* <0 .001], and vitamin K1/Triglycerides [*β* (95% CI): –0.22 (–40.97 to –8.71), *p* < 0.01] in Model 1; serum total MGP level was also significantly associated with age [*β* (95% CI): 0.33 (1.97–5.03), *p* <0 .001], sex (male) [*β* (95% CI): 0.34 (15.65–39.21), *p* <0.001] and vitamin K2 ≤ 0.05 ng/ml [*β* (95% CI): 0.15 (0.82–24.55), *p* <0 .05] in Model 2.

**Table 2.** Regression analyses of serum matrix Gla protein levels in all subjects (*n* = 152).

Variable	Univariate regression analyses			Multiple regression analysis					
				Model 1			Model 2		
	*β*	95% CI	*p*	*β*	95% CI	*p*	*β*	95% CI	*p*
Age (years)	0.30	1.59 - 4.86	<0.001	0.31	1.76 - 4.79	<0.0001	0.33	1.97 - 5.03	<0.001
Male	0.30	12.03 - 37.09	<0.001	0.27	10.41 - 34.06	<0.001	0.34	15.65 - 39.21	<0.001
Vitamin K1/Triglycerides (ng/mg)	–0.30	–50.33 - –16.09	<0.001	–0.22	–40.97 - –8.71	<0.01			
Vitamin K2 ≤ 0.05 ng/mL	0.11	–4.07 - 22.27	0.17				0.15	0.82 - 24.55	<0.05
Vitamin K2/Triglycerides (ng/mg)^a^	–0.03	–8.72 - 5.83	0.70						
Body mass index (kg/m^2^)	0.11	–1.06 - 5.13	0.20						

Model 1 included age, sex (male), and vitamin K1/Triglycerides, which exhibited significance in univariate analyses. Model 2 was nearly identical to model 1, but excluded vitamin K1/Triglycerides and included vitamin K2 ≤ 0.05 ng/ml.

^a^Values in subjects with measurable plasma vitamin K2 (24 HD patients and 14 healthy controls).

*β*: Standardized partial regression coefficient, CI: confidence interval, vitamin K1/Triglycerides: plasma levels of vitamin K1 corrected for triglycerides, vitamin K2/Triglycerides: plasma levels of vitamin K2 corrected for triglycerides.

Clinical characteristics in all MHD patients (*n* = 112), as well as in MHD patients stratified into three groups according to CACS [CACS < 100 (*n* = 26), CACS 100–399 (*n* = 23), and CACS ≥ 400 (*n* = 63)], are shown in Table 3. Of 112 MHD patients, 100 (89.2%) had CACS ≥1; mean ± SD of age was 51 ± 7 years, median (IQR) of CACS was 702 (109–2426) and median dialysis vintage was 88 (34–158) months. The presence of diabetes mellitus, presence of past or present CVD, presence of hypertension, active vitamin D3 use, phosphate binders use, calcium carbonate use, cinacalcet use, and statin use were observed in 44 (39.3%), 38 (33.9%), 81 (72.3%), 86 (76.8%), 101 (90.2%), 53 (47.3%), 34 (30.4%), and 23 (20.5%) patients. The patients with CACS ≥400 showed significantly older age, longer dialysis vintage, higher prevalence of CVD, higher intact parathyroid hormone (iPTH) level, lower serum magnesium level, higher C-reactive protein (CRP) level, and lower HDL cholesterol level, compared with patients with CACS <100 (*p* <0 .05). The patients with CACS 100–399 showed significantly higher iPTH levels than patients with CACS <100 (*p* <0 .05). Other parameters did not show significant differences among the three CACS groups.

**Table 3.** Clinical characteristics in all hemodialysis patients and in three groups stratified by CACS (CACS < 100, CACS 100–399, and CACS ≥400).

	All	CACS <100	CACS 100-399	CACS ≥400
Characteristics	*n* = 112	*n* = 26	*n* = 23	*n* = 63
CACS	702 (109–2426)	0 (0–16)	182 (118–361)	1908 (961–3378)
Age (years)	51 ± 7	47 ± 7*	49 ± 7	52 ± 5
Male [*n* (%)]	66/112 (58.9)	15/26 (57.7)	14/23 (60.9)	37/63 (58.7)
Dialysis vintage (months)	88 (34–158)	71 (23–124)**	90 (35–169)	116 (66–213)
Presence of diabetes mellitus [n (%)]	44/112 (39.3)	9 (34.6)	9 (39.1)	25 (41.3)
Presence of CVD [n (%)]	38/112 (33.9)	3/26 (11.5)**	6/23 (26.1)	29/63 (46.0)
Presence of hypertension [*n* (%)]	81/112 (72.3)	19 (73.0)	16 (69.6)	46 (73.0)
Vitamin K1/Triglycerides (ng/mg)	0.39 (0.24–0.70)	0.53 (0.31–0.91)	0.42 (0.21–0.68)	0.10 (0.04–0.14)
Vitamin K2 ≤ 0.05 ng/mL	88/112 (78.6)	19/26 (73.1)	18 (78.3)	51 /63 (81.0)
Matrix Gla protein (ng/mL)	288 ± 44	278 ± 43	291 ± 49	295 ± 38
Serum albumin (g/dL)	3.9 (3.6–4.0)	4.0 (3.6–4.1)	3.9 (3.8–4.3)	3.8 (3.6–4.0)
Albumin-adjusted serum calcium (mg/dL)	92 (8.7–9.7)	9.4 (8.9–9.9)	9.5 (8.9–9.8)	9.1 (8.7–9.6)
Serum phosphate (mg/dL)	5.6 ± 1.3	5.5 ± 0.1	5.6 ± 1.05	5.4 ± 1.2
Intact parathyroid hormone (pg/mL)	149 (88–220)	108 (68–151)*†	169 (112–231)	170 (98–259)
Serum magnesium (mg/dL)	2.4 ± 0.4	2.6 ± 0.4*	2.5 ± 0.4	2.4 ± 0.4
C-reactive protein (mg/dL)	0.10 (0.03–0.27)	0.03 (0.02–0.12)**	0.34 (0.05–9.49)	0.10 (0.04–0.32)
Total cholesterol (mg/dL)	156 (135–176)	150 (130–176)	165 (138–187)	160 (137–176)
HDL cholesterol (mg/dL)	53 (41–47)	58 (52–72)*	52 (39–73)	50 (37–62)
LDL cholesterol (mg/dL)	83 ± 26	77 ± 20	91 ± 32	52 ± 17
Triglycerides (mg/dL)	102 (63–150)	89 (49–132)	100 (48–193)	102 (68–150)
Active vitamin D3 use [*n* (%)]	86/112 (76.8)	20/26 (76.9)	17/23 (73.9)	49/63 (77.7)
Phosphate binders use [*n* (%)]	101/112 (90.2)	22/26 (84.6)	20/23 (87.0)	59/63 (93.7)
Calcium carbonate use [*n* (%)]	53/112 (47.3)	10/26 (38.5)	11/23 (47,8)	32/63 (50.8)
Cinacalcet use [*n* (%)]	34/112 (30.4)	7/26 (26.9)	6/23 (26.0)	21/63 (33.3)
Statin use [*n* (%)]	23/112 (20.5)	4/26 (15.4)	5/23 (21.7)	14/63 (22.2)

Values are expressed as means ± standard deviations or medians (interquartile ranges), as appropriate. All abbreviations are as defined in Table 2.

CACS: Agatston coronary artery calcium score, CVD: past and present cardiovascular disease (e.g. coronary artery disease, aortic aneurysms, cerebral infarction, cerebral hemorrhage, and/or peripheral artery disease), hypertension: predialysis blood pressure ≥140/90 mmHg, HDL: high-density lipoprotein, LDL: low-density lipoprotein.

*p < 0.05, **p < 0.01 compared with patients with CACS ≥400.

†*p* < 0.05, compared with patients with CACS 100–399.

Regression analyses were conducted for CACS in MHD patients (*n* = 112). Independent variables included in univariate analyses were all the variables in Table 3, with the exception of CACS. In univariate analyses, only age, dialysis vintage, presence of CVD, serum magnesium, HDL cholesterol level, and active vitamin D3 use were significantly associated with Log (CACS + 1) (*p* < 0.05) (Table 4, Additional file 1: Supplementary material Table S1). As shown in Table 4, in multivariate regression analyses for CACS in MHD patients, Model 1 included all variables that exhibited significance in univariate analyses, as well as the presence of diabetes, presence of hypertension, and vitamin K1/Triglycerides. Model 2 was nearly identical to Model 1, but included MGP level and excluded vitamin K1/Triglycerides. No associations were observed between Log (CACS + 1) and vitamin K1/Triglycerides (Model 1), or between Log (CACS + 1) and serum MGP level (Model 2). However, the following factors exhibited significant associations with Log (CACS + 1) in multivariate analysis of MHD patients (*p* < .05): age, dialysis vintage, presence of CVD, and HDL cholesterol level (Model 1, 2).

**Table 4.** Regression analyses for cardiovascular calcium score in hemodialysis patients (*n* = 112).

Variable	Univariate analyses			Multivariate analysis					
				Model 1			Model 2		
	*β*	95% CI	*p*	*β*	95% CI	*p*	*β*	95% CI	*p*
Vitamin K1/Triglycerides (ng/mg)	–0.19	–1.03 - 0.02	0.06	–0.09	–0.79 - 0.28	0.34			
Matrix Gla protein (ng/mL)	0.08	–0.00 - 0.01	0.41				0.08	–0.01 - 0.01	0.35
Age (years)	0.27	0.01 - 0.09	<0.01	0.19	0.00 - 0.07	<0.05	0.20	0.01 - 0.07	<0.05
Dialysis vintage (months)	0.26	0.00 - 0.01	<0.01	0.40	0.00 - 0.01	<0.0001	0.41	0.00 - 0.01	<0.0001
Presence of CVD	0.34	0.18 - 0.64	<0.001	0.24	0.09 - 0.50	<0.01	0.27	0.12 - 0.54	<0.01
Serum magnesium (mg/dL)	–0.23	–1.31 - –0.10	<0.05	–0.13	–0.93 - 0.13	0.14	–0.13	–0.93 - 0.13	0.14
HDL cholesterol (mg/dL)	–0.26	–0.03 - –0.00	<0.01	–0.21	–0.27 - –0.00	<0.05	–0.25	–0.03 - –0.01	<0.01
Active vitamin D3 use	–0.30	–0.60 - 0.13	<0.01	–0.14	–0.43 - 0.04	0.10	–0.15	–0.44 - 0.03	0.09
Presence of diabetes mellitus	0.18	–0.02 - 0.47	0.07	0.16	–0.04 - 0.45	0.10	0.15	–0.06 - 0.43	0.14
Presence of hypertension	0.11	–0.44 - 0.12	0.26	0.08	–0.35 - 0.12	0.35	0.10	–0.32 - 0.12	0.26
Vitamin K2 ≤ 0.05 ng/mL	0.57	–0.20 - 0.36	0.57						

All abbreviations are as defined in Tables 2 and 3. Hypertension: predialysis blood pressure ≥140/90 mmHg. Prior to regression analysis, cardiovascular calcium score (CACS) was transformed to Log (CACS + 1). In multivariate analysis, Model 1 included all variables that exhibited significance in univariate analyses, as well as the presence of diabetes, presence of hypertension, and Vitamin K1/Triglycerides. Model 2 was nearly identical to Model 1, but included matrix Gla protein and excluded Vitamin K1/Triglycerides.

Univariate logistic regression analyses for the presence of CVD were conducted with the same independent variables in Table 3, with the exception of the presence of CVD in MHD patients (*n* = 112). In univariate analyses, only vitamin K1/Triglycerides, serum MGP level, Log (CACS + 1), and serum albumin were significantly associated with presence of CVD (*p* < 0.05) (Table 5, Additional file 2: Supplementary material Table S2). As shown in Table 5, multivariate analysis for present and past CVD, Model 1 included all variables that exhibited significance in univariate analyses as well as the presence of diabetes and the presence of hypertension, but excluded MGP. Model 2 was nearly identical to Model 1, but excluded vitamin K1/Triglycerides and included MGP. Vitamin K1/Triglycerides was not significantly associated with the presence of CVD [Odds ratio (OR) 0.42, 95% CI (0.12–1.48), *p* = 0.18 ] (Model 1). However, serum MGP level was significantly associated with presence of CVD [OR 1.01, 95% CI (1.00–1.03), *p* <0 .05] (Model 2).

**Table 5.** Logistic regression analyses for presence of past or present cardiovascular disease in hemodialysis patients (*n* = 112).

	Univariate analyses			Multivariate analysis					
				Model 1			Model 2		
	OR	95% CI	*p*	OR	95% CI	*p*	OR	95% CI	*p*
Vitamin K1/Triglycerides (ng/mg)	0.43	0.16–1.16	<0.05	0.42	0.12–1.48	0.18			
Matrix Gla protein (ng/mL)	1.01	1.00–1.02	<0.05				1.01	1.00–1.03	<0.05
Log (CACS + 1)	2.13	1.38–3.62	<0.001	1.98	1.23–3.21	<0.01	2.04	1.24–3.36	<0.01
Serum albumin (g/dL)	0.04	0.00–0.39	<0.01	0.20	0.05–0.89	<0.05	0.28	0.07–1.17	0.07
Presence of diabetes mellitus	1.97	0.92–4.27	0.08	1.02	0.38–2.72	0.96	1.40	0.52–3.77	0.51
Presence of hypertension	1.67	0.69–4.04	0.25	1.76	0.60–5.17	0.30	1.86	0.62–5.51	0.27
Vitamin K2 ≤ 0.05 ng/mL	1.19	0.49–3.06	0.70						

All abbreviations are as defined in Tables 2 and 3.

OR: odds ratio, prior to logistic regression analysis, cardiovascular calcium score (CACS) was transformed to Log (CACS + 1). Hypertension: predialysis blood pressure ≥140/90 mmHg. In multivariate analysis for present and past cardiovascular disease, Model 1 included all variables that exhibited significance in univariate analyses as well as the presence of diabetes and the presence of hypertension, but excluded matrix Gla protein. Model 2 was nearly identical to Model 1, but excluded vitamin K1/Triglycerides and included matrix Gla protein.

## Discussion

It has been reported that vitamin K is needed to activate the calcification inhibitor MGP [2]. Emerging debate between vitamin K antagonist therapy and worsening of vascular calcification (and calciphylaxis); moreover, there is emerging interest in vitamin K supplementation for vascular calcification and calciphylaxis in HD patients [2,16,17]. We found significantly lower plasma vitamin K1 values, vitamin K1/Triglycerides and a tendency for increased frequency of plasma levels of vitamin K2 ≤ 0.05 ng/ml in MHD patients, as well as significantly higher serum total MGP levels, compared with healthy controls. The prevalence of coronary artery calcification (CACS ≥1) was 89.2% in our study, similar to the prevalence described in previous reports [18,19]. Serum total MGP was significantly associated with the presence of CVD, but not with CACS, in MHD patients in our study. However, CACS was significantly associated with age, dialysis vintage, presence of CVD and low HDL cholesterol in MHD patients in our study, which was consistent with the findings of previous reports [11,12,20]. Measuring vitamin K in plasma is difficult because of low circulating vitamin K levels and lipid interference. We measured vitamin K by HPLC with electrochemical detection using the method of Wakabayashi et al. [15]; this method has been reported to reduce the proportions of poor-resolution chromatograms in the plasma of dialysis patients, as demonstrated by increased concentrations of total cholesterol and triglycerides [15]. Furthermore, vitamin K levels were adjusted for triglycerides in this study. Plasma levels of vitamins K1 and K2 (when vitamin K2 levels were measurable) of healthy subjects in this study were similar to values determined by HPLC with electrochemical detection in previous reports of Japanese subjects [15,21], although we could not find a relevant reference regarding these values in the overall Japanese population.

Patients with CKD appear to be negatively affected by vitamin K deficiency for at least three reasons: reduced dietary intake; reduced expression and activity of vitamin K epoxide reductase enzymes that enable recirculation of vitamin K, thus increasing its tissue availability [4]; and phosphate binder use, which prevents vitamin K absorption in the gut [22]. It has also been reported that the administration of phosphate binders, active vitamin D, and calcimimetics may inhibit the progression of vascular calcification; however, these approaches remain controversial [23]. It is uncertain whether statins promote vascular calcification [24]. Importantly, use of medications, such as active vitamin D3, phosphate binders (including calcium carbonate), cinacalcet, or statins, was not a significant predictor for CACS or the presence of CVD in MHD patients in our study.

An imbalance of calcification promoters [e.g. bone morphogenetic protein (BMP)-2, 4, and 6; osteocalcin; bone sialoprotein; alkaline phosphatase; calcium; and phosphate] and inhibitors (e.g. MGP, osteopontin, osteoprotegerin, fetuin A, klotho, vitamin K, and magnesium) in CKD may cause development of vascular calcification [16,25]. The precise function of MGP has not been elucidated, but may include calcium crystal growth, blockage of bone morphogenetic protein (BMP)-2 and BMP-4 functions, and inhibition of vascular calcification [16,26]. Low levels of vascular calcification are present in predialysis CKD, and vascular calcification significantly increases in patients on dialysis [25].

A noninvasive biomarker for vascular calcification would be of great value; it may be important to determine whether MGP can serve as a biomarker for vascular calcification in HD patients. Some studies have reported significant correlations between MGP and vascular calcification in HD patients [8–10,16,27], while other studies have reported that there is no significant relationship between MGP level and vascular calcification [28–30]. A positive correlation has been reported between vascular calcification scores and dephosphorylated-uncarboxylated MGP in HD patients [8,27], although an inverse correlation has also been reported between CACS and uncarboxylated MGP in HD patients [9]. Notably, no correlation has been reported between CACS and uncarboxylated MGP in HD patients [28]. Consistent with our results, previous studies have shown that total MGP is not closely related with CACS in HD patients [29,30]. Fusaro et al. reported lower plasma vitamin K1 levels, lower plasma menaquinones (vitamin K2) levels, and increased levels of total MGP in HD patients, compared with healthy controls; they also found an association between the vitamin K system and vascular calcification in HD patients [31]. Thus, they suggested that total MGP may not constitute a good marker of vascular calcification [31]. Schlieper et al. reported that dephosphorylated, carboxylated MGP levels were lower in dialysis patients than in normal subjects, which increased risks of all-cause and cardiovascular mortality [10]. However, we did not measure dephosphorylated, carboxylated MGP levels. Our finding of vitamin K deficiency and increased levels of total MGP in MHD patients may contradict the findings of prior studies, which reported that vitamin K is needed to activate the vascular calcification inhibitor, MGP [2,7]. We presume that the increased levels of total MGP in our MHD patients may represent increased levels of inactive MGP and that increased levels of total MGP may be a risk factor for CVD, as we observed a significant association between the presence of CVD and total MGP levels in our study. An association between CACS and serum total MGP may not have been detected in our study because the measurement of overall serum MGP was performed without differentiation between uncarboxylated and carboxylated forms of MGP. We also suspect that the lack of an association between CACS and total MGP at baseline in our study does not exclude the possibility that persistently high total MGP level may influence CACS progression; this should be confirmed by additional studies. We only measured total serum MGP, rather than the individual MGP species; thus, our results further support the hypothesis that vitamin K is a cofactor that mediates the activation/conversion of MGP, and is not actively involved in the synthesis of MGP [2].

There are clearly complex relationships between calcification inhibitor proteins and CACS, which are influenced by clinical setting and dialysis vintage. Further investigation to dialysis vintage of various MGP species (e.g. total uncarboxylated MGP, dephosphorylated-uncarboxylated MGP, and dephosphorylated-carboxylated MGP) are needed to elucidate the specific effect of MGP on CACS in MHD patients.

Our study had several limitations. Its primary limitation was its cross-sectional design and inclusion of Japanese subjects alone; notably, there was heterogeneity among subjects with respect to CKD etiology and dialysis vintage. Additionally, we did not measure MGP species; rather, we measured total MGP, which limits conclusions regarding the roles of particular MGP species in the development of vascular calcification. Furthermore, we did not collect information regarding oral vitamin K1 and K2 intake among the subjects.

In conclusion, we propose that the end-stage renal disease on hemodialysis is a deficiency state of vitamin K based on current study. Total MGP was significantly higher in MHD patients compared to healthy subjects and total MGP was associated with the presence of CVD, but not CACS, in MHD patients.

## Supplementary Material

Supplemental Material

## Data Availability

The data are not available for public access because of patient privacy concerns, but are available from the corresponding author on reasonable request.
