# Supplementary material for: Relationship of matrix Gla protein and vitamin K with vascular calcification in hemodialysis patients
Source: Ren Fail. 2019 Sep 20;41(1):770–7. doi: 10.1080/0886022X.2019.1650065 (PMC7011966; doi:10.1080/0886022X.2019.1650065)
Supplement: Supplemental Material [file IRNF_A_1650065_SM3513.pdf]

Supplementary Table 1. Univariate regression analyses for cardiovascular calcium score in hemodialysis patients (n = 112)

Variable	Univariate analyses		
	β	95% CI	P
**Male**	**0.07**	**-0.22-0.44**	**0.51**
**Serum albumin (g/dl)**	**-0.16**	**-1.27-0.15**	**0.12**
**Albumin-adjusted serum calcium (mg/dl)**	**-0.15**	**-0.57-0.09**	**0.15**
**Serum phosphate (mg/dl)**	**-0.07**	**-0.28-0.13**	**0.49**
**Intact parathyroid hormone (pg/ml)**	**0.20**	**-3.83-0.00**	**0.06**
**C-reactive protein (mg/dl)**	**-0.14**	**-0.55-0.10**	**0.18**
**Total cholesterol (mg/dl)**	**0.01**	**-0.01-0.01**	**0.90**
**LDL cholesterol (mg/dl)**	**-0.04**	**-21.19-14.64**	**0.72**
**Triglycerides (mg/dl)**	**0.14**	**-0.01-0.01**	**0.17**
**Phosphate binders use**	**0.13**	**-0.16-0.72**	**0.21**
**Calcium carbonate use**	**0.10**	**-0.12-0.37**	**0.32**
**Cinacalcet use**	**0.03**	**-0.23-0.29**	**0.81**
**Statin use**	**0.10**	**-0.15-0.45**	**0.32**

β: Standardized partial regression coefficient, CI: confidence interval
LDL: low-density lipoprotein

Supplementary Table 2. Univariate logistic regression analyses for presence of past or present cardiovascular disease in hemodialysis patients (n = 112)

Variable	Univariate analyses		
	OR	95% CI	P
Age (yeas)	1.04	0.98-1.11	0.19
Male	2.34	0.72-7.59	0.16
Dialysis vintage (months)	1.00	0.99-1.00	0.93
Albumin-adjusted serum calcium (mg/dl)	1.07	0.62-1.85	0.81
Serum phosphate (mg/dl)	0.99	0.74-1.33	0.95
Intact parathyroid hormone (pg/ml)	1.00	1.00-1.01	0.09
Serum magnesium (mg/dl)	0.51	0.18-1.41	0.19
C-reactive protein (mg/dl)	1.58	0.74-3.38	0.24
Total cholesterol (mg/dl)	0.99	0.97-1.00	0.06
HDL cholesterol (mg/dl)	0.99	0.97-1.01	0.33
LDL cholesterol (mg/dl)	0.99	0.97-1.00	0.10
Triglycerides (mg/dl)	1.00	0.99-1.01	0.79
Active vitamin D3 use	0.77	0.31-1.92	0.58
Phosphate binders use	0.48	0.13-1.77	0.27
Calcium carbonate use	0.73	0.33-1.60	0.43
Cinacalcet use	0.90	0.39-2.13	0.82
Statin use	1.05	0.40-2.75	0.92

OR: Odds ratio, CI: confidence interval
HDL: high-density lipoprotein, LDL: low-density lipoprotein
